# Cutaneous Oncology: From Research to Diagnosis and Management

**DOI:** 10.1155/2016/7819428

**Published:** 2016-06-02

**Authors:** Ieva Saulite, Elisabeth Roider, Razvigor Darlenksi, Ahmad Jalili, Emmanuella Guenova

**Affiliations:** ^1^Department of Infectology and Dermatology, Riga Stradins University, Riga, Latvia; ^2^Department of Dermatology, Cutaneous Biology Research Center, Massachusetts General Hospital, Harvard Medical School, Charlestown, MA, USA; ^3^Department of Dermatology and Venereology, Tokuda Hospital Sofia, Sofia, Bulgaria; ^4^Section of Dermatology and Venereology, Trakia University, Stara Zagora, Bulgaria; ^5^Division of Immunology, Allergy and Infectious Diseases (DIAID), Department of Dermatology, Medical University of Vienna, Vienna, Austria; ^6^Department of Dermatology, University Hospital of Zurich, University of Zurich, Gloriastrasse 31, 8091 Zurich, Switzerland

Skin cancer incidence rates show a tendency for continuous increase. Along with this rise, cutaneous oncology maintains an extensive interest for clinicians and researchers as well as the pharmaceutical industry. This special issue highlights and discusses exciting implications in exploration of broad spectrum of rare and common conditions with respect to both diagnosis and treatment of cutaneous malignancies.

An important aspect concerning angiosarcoma, a highly aggressive malignant tumor that still lacks a common consensus for the treatment approach, has been targeted in the article titled “Surgical Treatment and Prognosis of Angiosarcoma of the Scalp: A Retrospective Analysis of 14 Patients in a Single Institution” by J. H. Choi et al. Although authors report a poor prognosis of angiosarcoma despite aggressive treatments, they demonstrate that in case of angiosarcoma sufficiently deep resection margins are essential for controlling local recurrence, further highlighting the need for studies aiming to evaluate multidisciplinary treatment modalities.

A well-structured study of a high clinical relevance “Comparison of the Clinical Characteristics and Outcome of Benign and Malignant Eyelid Tumors: An Analysis of 4521 Eyelid Tumors in a Tertiary Medical Center” by Y.-Y. Huang et al. provides essential demographic and clinical features to support differentiation between benign and malignant eyelid tumors. Furthermore applied treatment method and outcome in case of benign and malignant eyelid tumors are evaluated aiming to support clinical decision to reduce the esthetic disfigurement and morbidity especially in case of malignant eyelid tumors.

Improving aspects of early diagnosis of skin cancer remains a fundamental concern. The role of computer assistance for clinical decision with regard to detecting skin cancer needs to be defined and evidenced by appropriate studies. A profoundly actual up-to-date study “Computer-Aided Decision Support for Melanoma Detection Applied on Melanocytic and Nonmelanocytic Skin Lesions: A Comparison of Two Systems Based on Automatic Analysis of Dermoscopic Images” by K. Møllersen et al. compares capability of two clinical decision support computer systems to detect not only melanocytic but also nonmelanocytic skin lesions. Authors report potential of commercially available system* Nevus Doctor* being able to detect nonmelanocytic skin cancer along with remaining melanoma sensitivity and current limitations due to misclassification of benign nonmelanocytic lesions.

CD30+ lymphoproliferative disorder (LPD) is a rare variant of T-cell lymphoma that in some cases is recurrent and treatment resistant. Therefore for palliation of CD30+LPD to avoid unnecessary side effects of aggressive treatment approaches radiotherapy may be considered. However there is a lack of data endorsing radiotherapy (RT) in CD30+ LPD. M. S. Gentile et al. in the study titled “Single-Fraction Radiotherapy for CD30+ Lymphoproliferative Disorders” in a retrospective review prominently assess the clinical response of refractory or recurrent CD30+ lymphoproliferative disorder lesions to palliative radiation therapy suggesting palliative localized RT for symptomatic CD30+ LPD refractory or recurrent to other therapies. Study supports that palliative radiation therapy is an effective well tolerated treatment modality for CD30+ LPD refractory or recurrent to other therapies and reports single fraction of 750–800 cGy being as effective as a multifractionated course and more convenient.

In another rare malignancy, low-grade sarcoma, dermatofibrosarcoma protuberans (DFSP) margins of the tumor tend to extend beyond the macroscopic ones. Therefore the incomplete removal of the tumor leads to recurrences. This concern is accurately aimed in comprehensive clinical study titled “Wide Local Excision for Dermatofibrosarcoma Protuberans: A Single-Center Series of 90 Patients” by B. J. Kim et al. which evaluates efficacy of wide local excision for DFSP. Authors report that wide local excision with adequate lateral and deep margins can effectively control local recurrence rate and is a simple and effective method to treat DFSP.

Although most skin rashes in the neonatal age are benign, some may represent malignancy or be a feature related to other abnormalities. Therefore to provide the best possible outcome a considerable assessment and an adequate follow-up for each condition are of high value. A profoundly written educational review and a case series “The Skin as an Early Expression of Malignancies in the Neonatal Age: A Review of the Literature and a Case Series” by V. Mondì et al. comprehensively discuss the main tumor types presenting with a cutaneous involvement in neonates, additionally providing a collection of didactic cases presenting with an early skin manifestation of malignancies in illustrative case series.

A review article titled “Sézary-Syndrome and Atopic Dermatitis: Comparison of Immunological Aspects and Targets” by I. Saulite et al. provides an interesting perspective by comparing currently accepted immunological aspects and possible therapeutic targets in atopic dermatitis (AD) and Sézary syndrome (SS). Authors discuss clinical appearance and the hallmark immunological characteristics of SS highlighting some striking similarities with acute flares of atopic dermatitis (AD). Taking into consideration the overlap of several immunological features, the application of similar therapeutic approaches in certain stages of both diseases that may come into consideration is discussed.

Hence, with a definite commitment to improve every aspect contributing to skin cancer this special issue consisting of original articles and educational reviews aims to be translated in clinical advancements for patients with skin cancer.

*Ieva Saulite* *Elisabeth Roider* *Razvigor Darlenksi* *Ahmad Jalili* *Emmanuella Guenova*

